# Antepartum Computerized Cardiotocography in High-Risk Pregnancies: Comparative Analysis of Fetal Heart Rate Parameters in Hypertensive Disorders of Pregnancy, Diabetes and Intrahepatic Cholestasis

**DOI:** 10.3390/jcm15020720

**Published:** 2026-01-15

**Authors:** Bianca Mihaela Danciu, Anca Angela Simionescu

**Affiliations:** 1 Department of Obstetrics and Gynecology, Carol Davila University of Medicine and Pharmacy, 050474 Bucharest, Romania; bianca-mihaela.danciu@drd.umfcd.ro; 2 Department of Obstetrics, Gynecology and Neonatology, National Institute for Maternal and Child Health “Alfred Rusescu”-Polizu, 127715 Bucharest, Romania; 3 Department of Obstetrics and Gynecology, Filantropia Clinical Hospital, 050474 Bucharest, Romania

**Keywords:** computerized cardiotocography (cCTG), high-risk pregnancies, hypertensive disorders of pregnancy, gestational diabetes, intrahepatic cholestasis, fetal hypoxia

## Abstract

**Background/Objectives**: Antepartum computerized cardiotocography (cCTG) represents an essential tool for assessing fetal well-being. This study aimed to comparatively evaluate antepartum cCTG-derived indices across high-risk pregnancies to identify distinctive fetal autonomic and reactivity profiles. **Methods**: A comparative analysis of antepartum cCTG parameters was conducted. The cohort included pregnancies beyond 28 weeks of pregnancy, 169 cases of hypertensive disorders of pregnancy (HDP), 146 of gestational diabetes mellitus (GDM), 86 of intrahepatic cholestasis (ICP), and 87 low-risk pregnancies as controls. **Results**: Baseline FHR remained within the physiological range across all groups (110–160 bpm; *p* > 0.05). Dynamic cCTG parameters exhibited clear pathology-dependent alterations. Short-term variability (STV) showed a stepwise decline from controls to ICP and GDM, reaching its lowest values in HDP (mean 1.08 bpm; *p* < 0.00001), accompanied by an increased proportion of epochs with STV < 1 bpm. Long-term variability suppression (LTV < 5 bpm) was significantly higher in GDM and HDP (*p* = 0.0077). Acceleration frequency decreased across all pathological groups, with the most pronounced reduction observed in HDP, whereas fetal movements were paradoxically elevated in both GDM and HDP. Total decelerations were more frequent in ICP and HDP; however, repetitive, late, prolonged, and >5 min decelerations remained rare and did not differ significantly between groups. **Conclusions**: HDP showed the most unfavorable cCTG profiles, consistent with impaired fetal autonomic regulation and chronic subclinical hypoxemia. GDM and ICP had moderate changes, suggesting milder adaptive responses. These findings emphasize the value of quantitative cCTG in differentiating fetal autonomic patterns in high-risk pregnancies and the importance of tailored surveillance strategies.

## 1. Introduction

Fetal hypoxia remains a major contributor to perinatal morbidity and mortality, accounting for up to 50% of neonatal encephalopathy cases and 20–30% of perinatal deaths worldwide [1,2]. In high-resource settings, the incidence of term perinatal asphyxia is approximately 2 per 1000 births, whereas rates may be nearly ten-fold higher in regions with limited access to maternal and neonatal care [3,4]. Among affected neonates, 15–20% die during the early neonatal period, and up to one quarter of survivors develop long-term neurodevelopmental impairment [3], underscoring the need for accurate and early detection of fetal compromise.

In physiological pregnancies, fetal oxygenation is maintained through efficient placental exchange and well-coordinated autonomic regulation [5]. When oxygen availability begins to decline, the fetus activates a series of compensatory mechanisms, most notably the redistribution of blood flow toward vital organs and the modulation of autonomic cardiac control [5]. As oxygen availability further declines, these compensatory pathways progressively fail, resulting in metabolic acidosis, myocardial depression, reduced heart rate variability, and ultimately irreversible neurological and systemic injury [5,6,7]. These functional changes manifest early in cardiotocographic (CTG) and computerized CTG (cCTG) parameters, providing measurable indicators of altered fetal autonomic regulation.

Because most biophysical indicators of fetal distress become abnormal only at advanced stages of hypoxia [8], CTG and cCTG remain the primary tools for continuous antepartum and intrapartum surveillance. However, conventional CTG is constrained by subjective visual interpretation, substantial interobserver variability, and incomplete standardization across classification systems [9], despite recent efforts to harmonize international guidelines [10]. The resulting inconsistency limits the reliability of key visual indicators of hypoxia, such as reduced baseline variability, absent accelerations, and recurrent late decelerations [11,12]. cCTG was developed to overcome these limitations by introducing objective, quantitative, and reproducible indices, including short-term variability (STV) and long-term variability (LTV). These parameters allow detection of early autonomic dysfunction before abnormalities become apparent on conventional tracings, thereby enhancing sensitivity for evolving fetal compromise and supporting more consistent clinical decision-making [11,12,13,14]. cCTG is therefore a sensitive and reproducible tool for identifying evolving fetal compromise and guiding timely intervention [15,16,17].

Maternal comorbidities, including hypertensive disorders of pregnancy (HDP), gestational diabetes mellitus (GDM), and intrahepatic cholestasis of pregnancy (ICP), may alter placental perfusion, fetal metabolic environment, or exposure to bile acids, leading to characteristic modifications in CTG or cCTG patterns even in the absence of overt hypoxia. Although these conditions do not uniformly induce fetal distress, each can influence autonomic regulation through distinct pathophysiological pathways, resulting in deviations from the cardiotocographic profile expected in physiological pregnancies.

To address existing knowledge gaps, the aim of the present study was to compare cCTG parameters between a physiological control group and pregnancies complicated by HDP, GDM, and ICP in order to characterize condition-specific alterations in fetal autonomic behavior. These findings may advance our understanding of how maternal pathology shapes fetal surveillance metrics in cCTG and may better inform clinical decision-making in pregnancy care, ultimately contributing to improved maternal and neonatal outcomes.

## 2. Materials and Methods

The data analyzed in this study were collected as part of the first author’s doctoral research conducted at the Filantropia Clinical Hospital of Obstetrics and Gynecology, Bucharest, Romania, Carol Davila University of Medicine and Pharmacy. The investigation was carried out between January 2022 and January 2025 as part of a broader research initiative focusing on antepartum fetal monitoring and neonatal outcomes in high-risk pregnancies.

This retrospective observational study included 488 singleton pregnancies, comprising 87 uncomplicated controls and 401 high-risk cases. High-risk pregnancies were classified into three pathological groups: hypertensive disorders of pregnancy (HDP), gestational diabetes mellitus (GDM), and intrahepatic cholestasis of pregnancy (ICP). The HDP group comprised gestational hypertension, chronic or pre-existing hypertension, and preeclampsia, while the GDM group included both pre-existing type 1 or type 2 diabetes and gestational diabetes. For subgroup analyses, the GDM group was further subdivided into two distinct subgroups: diet-controlled diabetes and insulin-requiring diabetes. Likewise, the HDP group was subdivided into two clinically defined subgroups: non-preeclamptic hypertensive disorders, including chronic hypertension and gestational hypertension, and a second subgroup comprising pregnancies complicated by preeclampsia.

Hospital discharge records were initially screened to identify pregnancies documented with one of the predefined maternal conditions: HDP, GDM, or ICP. The complete medical records of all identified cases were subsequently reviewed to verify eligibility according to the predefined inclusion and exclusion criteria. Inclusion criteria included singleton pregnancy, gestational age ≥ 28 weeks at the time of cCTG recording, and delivery at the same institution, whereas exclusion criteria included multiple gestations, major fetal congenital anomalies, incomplete or inconsistent clinical data, and cCTG recordings with signal loss exceeding 20% or lasting less than 20 min.

Eligible cases were then cross-checked against the Omniview SisPorto Central Fetal Monitoring System to confirm correspondence between clinical records and antepartum cCTG registrations with respect to patient identity and recording date. Corresponding cCTG recordings were further screened to ensure that monitoring was performed antepartum, outside labor, with a minimum recording duration of 20 min and in accordance with predefined technical quality requirements. We consider for each patient the last cCTG registration before delivery was indicated. Only cases fulfilling all clinical, administrative, and technical criteria were included in the final study cohort. A dedicated study-specific database was constructed using exclusively fully verifiable and internally consistent cases, and all recordings were anonymized prior to analysis.

Computerized fetal monitoring was performed using the Omniview SisPorto Central Fetal Monitoring System (version 4.0.15), following a standardized antepartum protocol outside of labor. The system automatically extracted fetal heart rate (FHR) and uterine activity parameters, which were subsequently verified and integrated into the research database together with gestational and perinatal clinical information.

The initial dataset comprised continuous cCTG-derived parameters reflecting fetal physiological behavior. Recording integrity was assessed using signal loss (%), which was applied exclusively as an eligibility criterion. Recordings with signal loss exceeding 20% were excluded, and signal loss was not retained for subsequent analyses. Core fetal parameters included baseline FHR, number of accelerations, and fetal movements. Variability indices comprised average short-term variability (STV), the percentage of STV values < 1 bpm, and the percentage of long-term variability (LTV) values < 5 bpm, serving as quantitative markers of fetal autonomic regulation and oxygenation. Deceleration-related features included total decelerations, repetitive decelerations, late decelerations, prolonged decelerations, and decelerations lasting more than five minutes. Although uterine activity was recorded, it was not retained in the final analytical dataset, as all recordings were obtained outside labor and the study focused exclusively on fetal heart-rate–derived parameters.

Gestational age at the time of cCTG examination was included to contextualize fetal maturity across groups. All cohorts comprised pregnancies between 28 and 41 weeks of gestation, with most recordings obtained during the late third trimester (37–40 weeks). For comparative analyses, four datasets were defined: a physiological control cohort and three pathological cohorts (GDM, ICP, and HDP). To reflect shared physiological mechanisms, gestational and pre-existing diabetes were analyzed together under GDM, while pregnancy-induced hypertension, chronic hypertension, and preeclampsia were unified under HDP.

Quality control procedures were applied uniformly across all recordings to ensure retention of high-fidelity cCTG traces. Variable selection was guided by physiological rationale and supported by prior fetal monitoring research. STV and LTV were retained as core indices of fetal autonomic regulation and oxygenation, while baseline FHR, accelerations, decelerations, and fetal movements served as markers of fetal reactivity and adaptive capacity. Following this selection process, eleven continuous cCTG parameters were preserved for comparative analysis: baseline FHR, number of accelerations, fetal movements, percentage of STV values < 1 bpm, average STV, percentage of LTV values < 5 bpm, total decelerations, repetitive decelerations, late decelerations, prolonged decelerations, and decelerations lasting more than five minutes. Collectively, this curated feature set provided an integrative representation of both tonic and phasic components of fetal cardiovascular regulation, forming a physiologically coherent and statistically robust basis for subsequent comparative and predictive analyses of perinatal outcomes.

All cCTG recordings were performed under routine antepartum clinical conditions at our institution, outside of labor, with a minimum recording duration of 20 min, in accordance with institutional clinical practice guidelines. Examinations were conducted during daytime hours, with the mother positioned in a semi-recumbent or left lateral position. No pharmacological stimulation or sedation was administered prior to or during monitoring. Maternal fasting status and recent physical activity were not systematically recorded due to the retrospective nature of the study; however, recordings were obtained during routine clinical care and reflect standard real-world surveillance conditions across all study groups. We include for each patient the last cCTG monitoring before delivery was indicated.

Statistical analysis was conducted using an open access online platform (https://www.socscistatistics.com/, accessed on 1 October 2025). First, the normality of data distribution was confirmed for each parameter and each patient group by building the histograms and assessing their shape. Al histograms appeared bell shaped, without marked skewness or multimodality (data not included). The homogeneity of variance across groups was verified using Levene’s test. Next, one-way ANOVA was used to compare select variables across the four study groups, yielding F-statistics and associated *p*-values. Significant differences were determined at α = 0.05 and pairwise comparisons were made using post hoc Tukey–Kramer HSD multiple comparison test. This approach enabled a standardized evaluation of group-level differences in the selected cCTG parameters and supported the identification of physiologically meaningful patterns relevant to subsequent interpretation of results and formulation of study conclusions. For all data sets that were statistically significant from each other, effect sizes were calculated using Hedges’ g, which is the recommended indicator for unequal sample sizes. In terms of interpretation, g = 0.2 indicate a small effect, g = 0.5 a medium effect, and g = 0.8 a large effect.

The study was designed and reported in accordance with the Strengthening the Reporting of Observational Studies in Epidemiology (STROBE) guidelines.

## 3. Results

In total, 14,412 singleton pregnancies were identified during the study period, of which 401 met the inclusion criteria for high-risk pregnancy group and 87 for control group. These comprised 169 cases of HDP, 146 cases of GDM, 86 cases of ICP, and 87 uncomplicated pregnancies serving as the control group. All cCTG recordings were obtained antepartum, beyond 28 weeks of gestation, and fulfilled the required quality threshold of less than 20% signal loss, ensuring that the extracted parameters accurately reflected physiological fetal heart-rate dynamics.

The primary aim was to determine whether intrinsic cCTG parameters differ between physiological pregnancies and those affected by gestational pathologies, and to evaluate the extent to which these differences should be integrated into routine cCTG interpretation. Additionally, the study sought to explore whether distinct pregnancy-related pathologies exhibit specific cCTG-derived markers of hypoxic deterioration, reflecting varying degrees of chronic fetal compromise, a clinically relevant aspect considering the inherently limited adaptive and compensatory capacity of the fetus.

In the following sections, the cCTG parameters outlined above will be examined comparatively, including baseline FHR, fetal movements, accelerations, the short- and long-term variability indices, and the full spectrum of deceleration-related metrics.

### 3.1. Descriptive Characteristics

The four study groups: control, GDM, ICP, and HDP, were broadly comparable in terms of demographic and clinical characteristics, including maternal background variables and gestational age. Maternal age ranged from 16 to 48 years across all cohorts. All cCTG examinations were performed after 28 weeks of gestation. This homogeneous distribution of gestational maturity ensured that fetal heart-rate patterns were evaluated under similar developmental conditions, thereby increasing the robustness of the comparative analyses.

The dataset encompassed a comprehensive range of cCTG parameters reflecting fetal autonomic regulation, reactivity, and adaptive capacity. Assessed variables included the baseline FHR (bpm), the number of accelerations, and fetal movements. Variability indices comprised average STV (bpm), the percentage of time with STV values < 1 bpm and the percentage of LTV values < 5 bpm. Deceleration-related parameters were also quantified, including the total number of decelerations, repetitive decelerations, late decelerations, prolonged decelerations, and episodes lasting more than five minutes.

### 3.2. Baseline Fetal Heart Rate

Baseline fetal heart rate (FHR) reflects the intrinsic pacemaker activity of the fetal myocardium and is modulated by the balance between sympathetic and parasympathetic autonomic influences [18]. As a global indicator of fetal well-being, baseline FHR typically remains stable unless significant disturbances such as hypoxia, acidemia, infection, or structural anomalies alter autonomic function [19]. In many gestational pathologies, early stages of fetal compromise preferentially affect variability and reactivity rather than the basal rate itself, making baseline FHR a relatively conservative parameter in detecting subtle or chronic distress [13].

In the present study, baseline FHR values fell consistently within the physiological range of 110–160 bpm across all four groups, including pregnancies complicated by GDM, ICP, and HDP (Table 1). Mean values ranged narrowly between 135.4 bpm in the control group and 137.7 bpm in HDP pregnancies. Slightly higher median values were observed in the HDP and ICP groups (138 bpm). Values for all groups were within expected physiological variability, and all data had a small coefficient of variation (6–7%). No statistically significant differences were found across groups (f-ratio = 0.92, *p* = 0.43).

Furthermore, the effect of different sub-pathologies for the GDM and HDP groups was also evaluated. For GDM pregnancies there were no effects of diet-controlled vs. insulin requiring groups. For HDP pregnancies, which were divided into non-preeclampsia vs. Preeclampsia subgroups, statistical analyses showed that the preeclamptic patients has significantly higher FHR values than the control (139.18 ± 9.47 compared to 134.29 ± 13.98, *p* = 0.009). The effect size was small to moderate (g = 0.4).

Overall, these findings indicate that the minor intergroup variations observed represent normal physiological variability and do not suggest pathology-specific modulation of intrinsic fetal cardiac activity. This stability is expected from a physiological perspective, as baseline FHR typically remains preserved until late stages of fetal compromise; earlier autonomic dysregulation is more readily expressed through changes in STV and LTV or through pathological decelerations.

The absence of significant differences among groups underscores the limited sensitivity of baseline FHR for detecting early or moderate chronic hypoxic stress. This further supports the focus on dynamic cCTG parameters, particularly variability indices and deceleration patterns, as more informative markers for distinguishing physiological from high-risk pregnancies.

### 3.3. Fetal Movements Perceived by Mother During cCTG

Fetal movements are a key indicator of fetal neurobehavioral integrity and autonomic reactivity, and their documentation during cCTG provides a valuable complement to the other physiological parameters assessed. In this study, fetal movements were manually registered by the mother during the cCTG monitoring, who pressed the event marker each time a movement was perceived. Consequently, these data reflect maternal perception at the time of monitoring and are subject to inter-individual variability and reporting bias. Therefore, findings related to fetal movements in general should be interpreted with caution and considered supportive rather than definitive indicators of fetal autonomic function.

**Figure 1:**
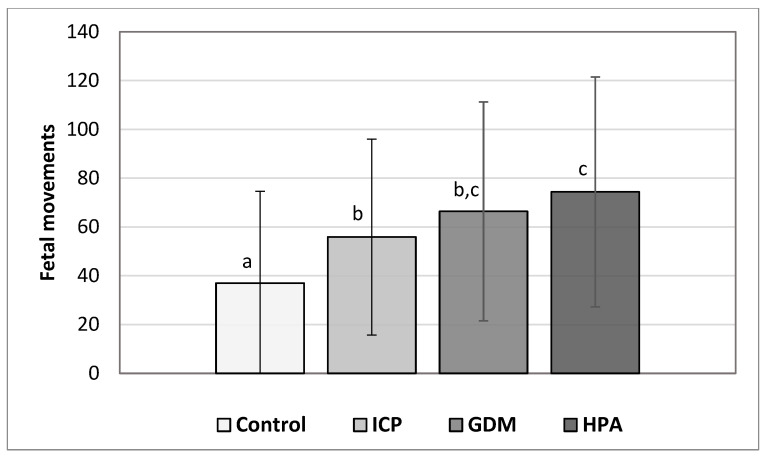
Fetal movements perceived by mother during cCTG monitoring for the Control group (N = 87), and ICP (N = 68), DGM (N = 146) and HPA (N = 169) groups. Values represent means for each group and error bars represent ±1 SD. Values not connected by the same letter are statistically different from each other (*p* < 0.05).

Across all groups, the number of maternally perceived movements recorded during cCTG was higher in pathological pregnancies compared with physiological controls (Table 2 and Figure 1). Mean counts increased progressively from 37.0 in the control group to 55.9 in ICP, 66.4 in GDM, and 74.5 in HDP pregnancies, with median values showing the same pattern. Variability was high across all cohorts (COV > 60%), reflecting physiological fluctuations related to behavioral-state cycling and intrinsic movement variability.

Despite this high variability, group comparisons revealed statistically significant differences in fetal movement counts by mothers among the study cohorts. All pathological groups demonstrated significantly higher movement counts compared with controls, with the highest values observed in pregnancies complicated by GDM and HDP. Compared with the Control group, the ICP pathology had a medium effect (g = 0.49) on fetal movement, GDM pathology had a medium-to-large effect (g = 0.70), and HDP had a large effect (g = 0.85). Direct comparison between HDP and ICP pregnancies yielded a moderate effect size (g = 0.41), indicating a clinically meaningful difference in perceived fetal activity between these pathological conditions.

**Figure 2:**
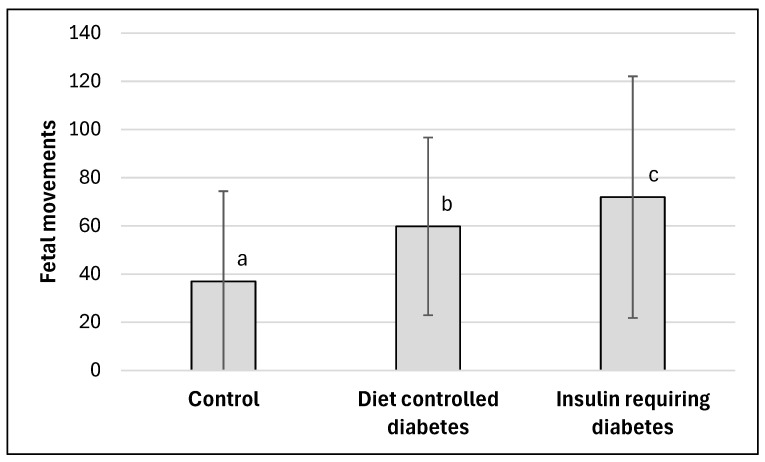
Fetal movement for Control (N = 87) compared to diet-controlled diabetes (N = 68) and insulin requiring diabetes (N = 79) groups. Values represent means for each group and error bars represent ±1 SD. Values not connected by the same letter are statistically different from each other (*p* < 0.05).

For GDM and HDP, different sub-pathologies also affected fetal movement values. For the diabetic pregnancies, both diet controlled and insulin requiring subgroups had significantly higher movements than the Control group (*p* < 0.05), but the latter had significantly higher values of the fetal movement than both the Control and the diet control groups (Figure 2). Diet-controlled had a moderate effect (g = 0.61) compared to the control, while the insulin requiring group had a large effect (g = 0.79). The two diabetic subgroups were also significantly different from each other (*p* < 0.05), and the effect of the specific pathology was small (g = 0.27).

**Figure 3:**
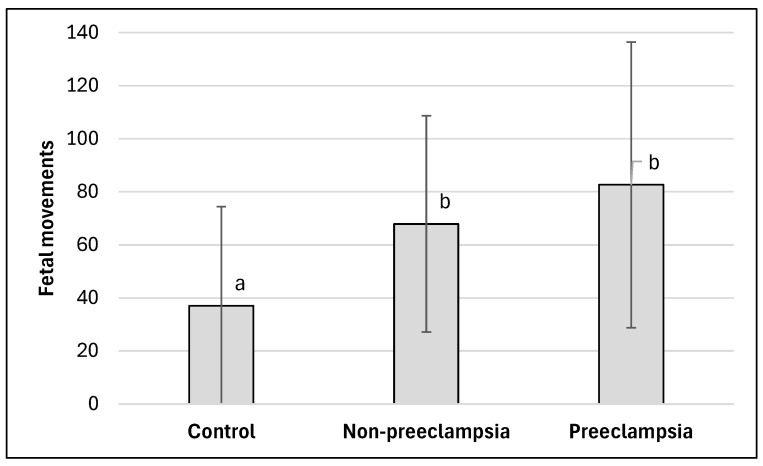
Fetal movement for Control (N = 87) compared to HDP non-preeclampsia (N = 97) and Preeclampsia (N = 71) groups. Values represent means for each group and error bars represent ±1 SD. Values not connected by the same letter are statistically different from each other (*p* < 0.05).

For HDP pregnancies, two sub-pathologies were identified: non-preeclampsia and Preeclampsia. Vales for both sub-pathologies were significantly different than the Control, but they were not significantly different from each other (see data in Figure 3). While both showed strong effect sizes, the effect for Preeclampsia was stronger (g = 1) than for non-preeclampsia (g = 0.79).

Given the lack of reporting in the existing literature regarding movement frequency during cCTG monitoring in HDP or GDM pregnancies, these results must be interpreted with caution. Their primary value is hypothesis-generating, suggesting that maternally perceived movement frequency may vary across different pathological contexts and may interact with concurrent cCTG features such as variability and accelerations. The observation that pregnancies complicated by HDP and GDM demonstrated higher movement counts raises the possibility that increased activity may, in some cases, reflect transient episodes of hypoxia-induced autonomic dysregulation or heightened fetal reactivity preceding decompensation, rather than simply representing preserved or enhanced fetal well-being.

Taken together, these findings suggest that increased maternally perceived fetal movements in high-risk pregnancies should be interpreted within the broader physiological and clinical context, particularly when accompanied by altered variability or reduced acceleratory responses. They should not be considered in isolation, as elevated movement frequency may represent an early and currently underrecognized sign of intrauterine stress in certain pathological states. Further research integrating both maternal perception and objective movement assessment is needed to clarify the mechanisms underlying this pattern and to determine whether such movement profiles possess predictive value for fetal adaptation or subsequent neonatal outcomes.

### 3.4. Short-Term Variability Indices

Short-term variability (STV) reflects beat-to-beat modulation of FHR and is widely recognized as one of the most sensitive cCTG-derived markers of fetal autonomic integrity and oxygenation status. In the present cohort, both STV-derived parameters, the percentage of epochs with STV < 1 bpm and the mean STV value, showed a consistent and physiologically coherent pattern across groups (Table 3 and Table 4). Compared with the control group (53.7%), the proportion of low-variability epochs was higher in each pathological cohort, measuring 57.4% in ICP, 58.5% in GDM, and 62.8% in HDP. Median values followed the same pattern. Coefficients of variation were relatively narrow (approximately 20%), indicating stable group-level trends despite inherent physiological variability.

Standardized effect size analysis using Hedges’ g revealed a small-to-moderate effect when comparing GDM with controls (g = 0.39) and a large effect for HDP relative to controls (g = 0.77). A similarly large effect size was observed when comparing HDP with ICP pregnancies (g = 0.77), indicating a substantial additional suppression of STV in HDP beyond that seen in ICP.

When data for diabetic pregnancies was analyzed for to the previously described subgroups, similar to STV < 1 bpm, only the diet-controlled diabetic subgroup showed a significant effect. The mean value for diet-controlled subgroup was statistically different from the Control group (*p* < 0.05), but not from the insulin-requiring subgroup; the effect size was moderate (g = 0.47). For HDP pregnancies, there were no statistically significant differences between the two subgroups, and each had a large effect size (g = 0.75) for the non-preeclamptic group vs. 0.78 for the preeclamptic group.

A similar gradient was observed for average STV values (Table 4). Mean STV declined from 1.35 bpm in controls to intermediate values in ICP and GDM (1.22 and 1.20 bpm, respectively), with the lowest mean recorded in HDP pregnancies (1.08 bpm). Coefficients of variation were comparable across cohorts, supporting the internal consistency of these findings. Collectively, these results indicate reduced fetal beat-to-beat variability in high-risk pregnancies relative to physiological controls.

Effect size estimates for mean STV further quantified these group separations. Compared with controls, mean STV showed a small-to-moderate reduction in GDM pregnancies (Hedges’ g = 0.38) and a large reduction in HDP pregnancies (Hedges’ g = 0.73). The difference between HDP and ICP pregnancies corresponded to a small-to-moderate effect size (Hedges’ g = 0.38), indicating a clinically meaningful but less pronounced separation between these pathological groups.

When analyzing the same previously mentioned subgroups, only the diet-controlled diabetic group showed a significant effect. Mean values were statistically different from the Control group (*p* < 0.05), but not from the insulin-requiring subgroup, with a moderate effect size (g = 0.45). For HDP pregnancies, there were no statistically significant differences between the two subgroups. They were each significantly smaller than the Control, and the effect size was large: g = 0.73 for the non-preeclamptic group vs. 0.66 for the preeclamptic group.

Despite the relatively homogeneous dispersion of values (coefficients of variation of approximately 20% across groups), statistical analysis revealed significant differences in both STV-derived indices. One-way ANOVA demonstrated robust intergroup differences for the proportion of epochs with STV < 1 bpm (F = 11.15, *p* < 0.00001) and for mean STV values (F = 9.78, *p* < 0.00001). As indicated by the different superscript letters in Table 3 and Table 4, all pathological cohorts exhibited significantly greater STV suppression compared with the control group, with the most marked reductions observed in HDP. These findings are consistent with established pathophysiological mechanisms, whereby uteroplacental insufficiency, intermittent hypoxemia, and chronic vascular or metabolic stress impair fetal autonomic responsiveness [20].

### 3.5. Accelerations

Accelerations represent a key marker of fetal reactivity and intact autonomic responsiveness, reflecting the ability of the fetus to mount brief, sympathetic–parasympathetic–mediated heart rate rises in response to movement or external stimuli [21]. In this cohort, acceleration counts were consistently lower in all pathological groups compared with physiological controls (Table 5). The control group demonstrated the highest mean number of accelerations (6.78; median 6), whereas reduced values were observed in ICP (mean 5.56; median 4) and GDM (mean 6.12; median 5). The most pronounced reduction occurred in HDP, where the mean number of accelerations declined to 4.21 (median 3), accompanied by the highest proportion of recordings without accelerations (15%, compared with <10% in all other groups).

Despite the wide inter-individual dispersion (COV 73–114%), the distribution of values across cohorts reveals a clear and progressive attenuation of fetal reactivity, most marked in HDP. This pattern aligns with established pathophysiological mechanisms, such as chronic uteroplacental insufficiency, increased placental resistance, and reduced oxygen reserve, that can blunt autonomic responsiveness and limit the capacity to generate accelerations, a hallmark of preserved fetal well-being.

Standardized effect size analysis using Hedges’ g quantified the magnitude of these differences. Compared with physiological controls, HDP pregnancies showed a moderate effect size reduction in acceleration frequency (Hedges’ g = 0.53). Direct comparison between HDP and GDM pregnancies yielded a small-to-moderate effect size (Hedges’ g = 0.38), indicating a meaningful additional reduction in fetal reactivity associated with HDP beyond that observed in GDM.

When analyzing the same previously mentioned subgroups, no significant effects were observed for the number of accelerations, with no statistically significant differences between diabetic subgroups or compared with the Control group. For HDP pregnancies, there were no statistically significant differences between the two subgroups. They were each significantly smaller than the Control, and the effect size was moderate: g = 0.54 for the non-preeclamptic group vs. 0.49 for the preeclamptic group.

Statistical analysis confirmed that these intergroup differences were significant. One-way ANOVA yielded an F-ratio of 5.60 with a *p*-value of 0.000879, demonstrating that the downward trend in acceleration frequency is unlikely to be attributable to random variability. As indicated by the different superscript letters in Table 6, controls displayed the highest level of reactivity, ICP and GDM showed intermediate values, and HDP consistently exhibited the lowest acceleration frequencies. The broader dispersion in the HDP group suggests greater physiological instability and supports the interpretation that these fetuses operate closer to the limits of their autonomic compensatory capacity.

### 3.6. Long Term Variability

Long-term variability (LTV) reflects slower oscillatory components of fetal heart-rate modulation and is influenced by central autonomic integration and fetal behavioral-state cycling. Periods during which LTV falls below 5 bpm are considered markers of diminished global reactivity and reduced adaptive capacity [22]. In the present cohort, all pathological groups demonstrated higher percentages of time with LTV < 5 bpm compared with physiological controls (Table 6). Mean values increased from 2.8% in the control group to 3.4% in ICP, 5.4% in GDM, and 6.8% in HDP, with median values remaining low across all cohorts due to the episodic nature of LTV suppression. Notably, the more substantial increases observed in the GDM and HDP group clearly distinguished these cohorts from the controls, despite the high coefficients of variation (161–222%) inherent to long-range variability metrics.

Standardized effect size analysis using Hedges’ g indicated a moderate effect size when comparing HDP pregnancies with physiological controls (g = 0.43), confirming that the observed increase in LTV suppression in HDP represents a clinically meaningful difference rather than a trivial shift driven by dispersion alone. Although effect size estimates were not calculated for all intergroup comparisons, the magnitude of separation between controls and the diabetic and HDP cohorts supports the presence of a true physiological gradient.

In subgroup-stratified analyses based on the previously described classifications, no statistically significant differences were observed between diabetic subgroups or between each subgroup and the Control. For HDP pregnancies, there were no statistically significant differences between the two subgroups, although they were both statistically larger than the control (*p* = 0.00002 and *p* = 0.00003, respectively). The accelerations averages for each subgroup were significantly larger than the Control, and the effect size was small (g = 0.11) for both subgroups.

Statistical analysis confirmed that the differences observed among groups were significant, indicating that the increased burden of LTV suppression in the GDM and HDP cohorts reflects a true physiological distinction rather than random variation. As shown by the distribution of values and the separation of means in Table 7, pregnancies complicated by GDM and HDP exhibited consistently longer periods of reduced LTV, whereas controls clustered near the lower end of the range. These findings are physiologically plausible, as both metabolic dysregulation and placental vascular dysfunction are known to impair central autonomic modulation and reduce the fetus’s ability to sustain long-range oscillatory patterns.

### 3.7. Decelerations

Decelerations represent transient reductions in FHR and encompass a broad physiological spectrum, from benign, movement-related vagal dips to patterns more suggestive of impaired oxygenation. In cCTG output, all of these events are grouped under the general label “decelerations,” a category that inherently combines heterogeneous phenomena and therefore requires cautious interpretation. Many non-pathological dips arise from fetal movements or transient cord compression, whereas more concerning patterns reflect altered autonomic regulation or reduced placental reserve [23].

Recording duration is another important methodological factor: longer traces increase the likelihood of capturing sporadic decelerations, many of which lack clinical relevance. In the present study, recordings were uniformly between 20 and 30 min, ensuring technical consistency while minimizing duration-related bias. For this reason, analyses focused on the presence versus absence of decelerations rather than raw event counts. Within this framework, total decelerations occurred more frequently in pathological pregnancies than in physiological controls (Table 7). The proportion of recordings containing at least one deceleration was lowest in the control cohort (20%) and increased in ICP (44%), GDM (36%), and HDP (50%). Mean values demonstrated a similar trend, rising from 0.31 in controls to 1.09 in ICP, 0.92 in GDM, and 1.19 in HDP. Although variability was substantial, an expected finding given the episodic nature of decelerations, the distribution suggests that pathological intrauterine conditions, particularly HDP, predispose fetuses to more frequent transient reductions in heart rate.

Statistical comparisons confirmed that total decelerations were significantly more frequent in ICP and HDP relative to controls, indicating that these cohorts exhibit increased susceptibility to transient vagal or hypoxemic stimuli even in the absence of overt fetal distress. No significant differences emerged for GDM, likely reflecting a broader range of mechanisms underlying deceleration generation in this group.

When decelerations were further categorized into clinically meaningful subtypes: repetitive, late, and prolonged, these events remained infrequent across all study groups. Repetitive decelerations occurred in only 1–5% of recordings (Table 8), with mean frequencies near zero, consistent with their sporadic character in the antepartum setting. Late decelerations, traditionally considered markers of uteroplacental insufficiency, were similarly uncommon, appearing in only 5–7% of recordings in each group (Table 9). This low frequency aligns with outpatient monitoring conditions, where uterine contractions, one of the primary triggers for late decelerations, are largely absent.

When stratified according to the previously described subgroups, only the insulin-requiring diabetic subgroup showed a significant effect. Mean values were statistically different from the Control group (*p* < 0.05), but not from the diet-controlled subgroup, with a small to moderate effect size (g = 0.38). For HDP pregnancies, there were no statistically significant differences between the two subgroups, but their averages were statistically larger than the control for both subgroups (*p* = 0.023 and *p* = 0.00008, respectively). The effect size was moderate (g = 0.48) for the non-preeclamptic subgroup and moderate to large (g = 0.70) for the preeclamptic subgroup.

Prolonged decelerations lasting more than 2 min were rarer still, occurring in only 1–4% of cases (Table 10). Their scarcity underscores that such events are typically associated with intrapartum dynamics rather than stable antepartum surveillance. Decelerations lasting more than 5 min were exceedingly rare across the entire cohort, observed in only 1–2% of recordings (Table 11). This extremely low incidence supports the notion that prolonged, clinically concerning decelerations are unusual in the absence of labor-related stressors. Importantly, the rarity of these prolonged patterns reinforces the principle that isolated decelerations, particularly those not meeting criteria for late, repetitive, or prolonged forms, should be interpreted in conjunction with variability and acceleratory indices rather than considered in isolation.

Although the descriptive analysis identified differences in the overall frequency of decelerations between groups, all clinically significant subtypes, including repetitive, late, and prolonged decelerations and those exceeding 5 min, were rare across the entire cohort. Because these events occurred in only 1–7% of recordings, the study lacks sufficient statistical power to determine whether such patterns differ meaningfully between pathology groups. Consequently, any apparent trends in severe deceleration types should be interpreted with caution, as the very low event rate limits the reliability of subgroup comparisons and precludes robust inference regarding their independent clinical relevance.

## 4. Discussion

The present study provides a detailed comparative evaluation of antepartum cCTG parameters in high-risk complicated pregnancies by HDP, GDM, and ICP in physiological pregnancies and in low-risk gestations. By analyzing 488 high-quality recordings obtained in the late second and third trimesters, the study delineates a clear, pathophysiologically coherent pattern of fetal autonomic alteration across these maternal conditions. The findings build upon, and extend, existing evidence suggesting that dynamic cCTG indices, especially STV, LTV, and acceleration frequency, offer superior discriminatory value compared with baseline FHR when assessing subtle or evolving fetal compromise in high-risk pregnancies.

Consistent with the observations of Buscicchio et al. (2010) [24], both GDM and HDP disorders exhibited measurable reductions in STV and an increased burden of LTV epochs. These alterations reflect an overall blunting of fetal autonomic responsiveness, a physiological hallmark of chronic metabolic or vascular stress [24]. The present study further demonstrates that these changes are not uniform across conditions: rather, they form a hierarchical gradient, with HDP pregnancies displaying the most profound impairment, GDM and ICP showing intermediate alterations, and physiological controls demonstrating the most preserved variability and reactivity.

The stability of baseline FHR across all four cohorts constitutes a central finding of this investigation. Mean values remained tightly clustered between 135 and 138 bpm, with narrow coefficients of variation and no statistically significant intergroup differences. This uniformity underscores the well-established principle that baseline FHR is a relatively conservative parameter, one that typically remains preserved until late or decompensated stages of fetal distress. Prior evidence has consistently shown that early autonomic dysregulation manifests first through changes in variability, reactivity, and episode architecture rather than through shifts in basal rate. This concept is well supported in physiological literature and reaffirmed by contemporary reviews, which emphasize that baseline FHR lacks sensitivity for detecting mild or moderate autonomic compromise [25]. Thus, the current data corroborate the understanding that baseline FHR, though clinically central, is insufficient as an early warning marker in high-risk pregnancies.

In contrast to baseline FHR, STV metrics demonstrate pronounced and statistically robust differences across groups. Both the proportion of epochs with STV < 1 bpm and the average STV value revealed a graded, pathology-dependent decline in beat-to-beat modulation. HDP exhibited the most severely altered profile, with a mean of 62.8% low-variability epochs and an average STV of 1.08 bpm, findings that reflect significant autonomic dampening. This pattern is pathophysiologically plausible. HDP are characterized by chronic uteroplacental malperfusion, increased placental resistance, endothelial dysfunction, oxidative stress, and altered neurohumoral signaling. These processes impair oxygen delivery and disrupt fetal autonomic circuits responsible for moment-to-moment modulation of heart rate. Previous studies using Doppler velocimetry, fetal ECG, and cCTG have demonstrated similar patterns of diminished reactivity and reduced autonomic reserve in fetuses exposed to HDP [26]. The concordance between present findings and prior literature [27] further validates the mechanistic interpretation that STV is among the earliest and most sensitive biomarkers of impaired fetal adaptive capacity.

GDM and ICP formed the expected intermediate tier, with moderate but significant reductions in STV relative to controls. In GDM, these changes likely reflect episodic hyperglycemia, metabolic instability, and increased oxidative stress, all of which influence autonomic maturation even in well-controlled pregnancies [28]. ICP exhibited milder reductions, consistent with experimental evidence demonstrating bile acid–mediated electrophysiologic effects on fetal myocardial conduction and calcium handling [29]. The present quantitative results support the notion that cCTG may detect autonomic disturbances in ICP earlier than conventional CTG [30,31]. These findings should be interpreted in the context of the limited literature addressing cCTG in ICP. Poncelet et al. reported no significant association between maternal serum bile acid concentrations, STV, and perinatal outcome, likely reflecting the use of STV as an isolated parameter in a small cohort with favorable outcomes and a low incidence of hypoxic events. The present study strengthens and extends these observations by demonstrating that, although STV alterations in ICP may appear modest in isolation, their clinical relevance becomes evident when interpreted within a broader multiparametric cCTG framework incorporating LTV suppression and reduced acceleratory activity [32].

LTV indices provided additional insight into the integrity of slower oscillatory components of fetal autonomic function. The proportion of epochs with LTV < 5 bpm was minimal in the control group and increased across all pathological cohorts. Among these, HDP exhibited the highest burden of LTV suppression, followed by GDM, indicating more pronounced long-range autonomic impairment in these groups. LTV reflects central autonomic regulation, fetal behavioral-state cycling, and integration within long-range regulatory loops. Therefore, increased LTV suppression complements STV reductions by indicating that both rapid and slow autonomic modulation are affected.

The parallel between high STV suppression and high LTV suppression in HDP suggests a global impairment of autonomic adaptability consistent with chronic or intermittent hypoxemia. The fact that both GDM and HDP demonstrated clinically meaningful LTV suppression aligns with known pathophysiologic mechanisms: vascular dysfunction in HDP and metabolic dysregulation in GDM can both impair central autonomic pathways and reduce the fetus’s capacity to sustain long-range oscillatory variability [33].

Acceleration frequency demonstrated a clear, coherent decline when comparing physiological with pathological pregnancies. Control fetuses showed the highest number of accelerations, whereas all three pathological groups exhibited reduced reactivity, with the most substantial attenuation observed in HDP, which also had the largest proportion of recordings lacking accelerations entirely. Because accelerations reflect intact sympathetic–parasympathetic interplay and adequate fetal oxygenation, their reduction provides further evidence of impaired autonomic responsiveness in these fetuses. In GDM and ICP, acceleration counts were moderately but consistently lower than in controls, mirroring the intermediate degree of autonomic alteration identified through STV and LTV indices. The close alignment between diminished accelerations, reduced STV, and a higher burden of low-variability epochs underscores the internal coherence of these findings and indicates that these parameters capture complementary aspects of the same underlying process, progressive autonomic compromise across high-risk pregnancies, most pronounced in HDP.

A key observation is that cCTG-detected fetal movement counts were consistently higher in all high-risk groups relative to the normal pregnancy cohort. This pattern diverges from traditional clinical teaching, which predominantly associates reduced fetal movements with deterioration of fetal condition [34]. The interpretation of this finding requires caution, especially in light of the heterogeneous and sometimes contradictory evidence available in the literature. In GDM, prior studies have not consistently demonstrated increased fetal motor activity; some report no significant differences in movement frequency [35], whereas others describe transient fluctuations around 28–32 weeks of gestation, a period characterized by rapid maturation of fetal neurobehavioral circuitry [36]. For HDP, classical work has historically linked decreased movements to placental insufficiency [37,38,39,40,41], while more recent research exploring autonomic dysregulation suggests that fetal motor output may vary unpredictably depending on the balance between sympathetic activation, behavioral-state instability, and intermittent perfusion fluctuations [42].

Within this context, the elevated movement frequency observed in our high-risk cohorts should not be interpreted as pathological hyperactivity or as a marker of improved fetal condition. Instead, when considered alongside reduced variability and blunted acceleratory patterns, particularly evident in HDP, the finding may reflect a form of autonomic disequilibrium rather than enhanced fetal vigor. This interpretation is consistent with publications indicating that episodes of increased fetal motor activity may arise in the setting of neurobehavioral imbalance rather than physiological robustness and may sometimes prompt unnecessary clinical intervention without clearly reflecting improved oxygenation [35]. As current guidelines do not emphasize increased movement patterns and focus almost exclusively on reduced fetal activity, the present findings should be viewed as hypothesis-generating and underscore the need for prospective studies designed to elucidate the physiological and prognostic significance of heightened fetal movement frequency within the broader cCTG context.

The analysis of decelerations revealed nuanced findings. Although total decelerations were significantly more common in ICP and HDP, subtype-specific patterns—repetitive, late, prolonged decelerations and decelerations lasting >5 min—were infrequent across all cohorts, and no significant differences were detected. This distinction is important for clinical interpretation. Antepartum cCTG often captures benign or non-pathological decelerations arising from fetal movements or transient cord compression [43]. The increased frequency of decelerations in HDP and ICP may therefore reflect heightened susceptibility to transient vagal activation or fluctuating perfusion rather than sustained hypoxemia. Moreover, the very low number of late or prolonged decelerations limits the ability of this study to draw firm conclusions regarding their independent clinical significance. These rare events should thus be interpreted with appropriate caution, as the sample does not provide adequate statistical power to fully characterize their prognostic value.

The overall pattern suggests that decelerations should not be interpreted in isolation but rather in conjunction with STV, LTV, and acceleration dynamics. It is likely that the composite signature—reduced variability, diminished acceleratory activity, and increased frequency of any decelerations—captures impaired autonomic adaptability more accurately than any single parameter alone, particularly in HDP. This integrative approach aligns with established physiological models of fetal stress response and underscores the value of multi-parameter assessment in antepartum surveillance.

Taken together, the results reveal a clear and physiologically coherent hierarchy of fetal autonomic impairment across the studied groups. HDP exhibited the most profoundly altered cCTG profile, characterized by markedly reduced STV, a greater burden of LTV suppression, fewer accelerations, and a higher frequency of decelerations. Although fetal movement counts were also elevated in HDP, the clinical significance of increased fetal activity remains uncertain; current evidence does not establish whether heightened movement represents a compensatory response, a manifestation of transient autonomic instability, or reflects a benign variation in fetal behavioral patterns. This constellation of abnormalities nonetheless indicates sustained autonomic dysregulation and chronic subclinical hypoxemic stress, suggesting that fetuses exposed to HDP may operate closer to the limits of their compensatory capacity.

GDM and ICP formed an intermediate tier, each showing consistent but less pronounced deviations from physiological patterns. Their cCTG profiles nonetheless indicate measurable autonomic alterations attributable to metabolic dysregulation in GDM and to bile-acid–mediated electrophysiological effects in ICP. In contrast, physiological pregnancies demonstrated preserved variability, reactivity, and autonomic balance, reinforcing their role as an appropriate reference group. Across all comparisons, dynamic cCTG indices, particularly those derived from variability and reactivity, clearly outperformed baseline FHR, underscoring the superior sensitivity of algorithm-derived metrics in detecting early signs of fetal autonomic imbalance.

Importantly, the present findings should be interpreted within the broader context of how fetal heart rate monitoring is currently applied in clinical practice. Accumulating evidence indicates that conventional visual interpretation of CTG is affected by substantial intra- and inter-observer variability, particularly when clinicians are required to classify traces as suspicious or pathological. An integrative review by Lukhele et al. demonstrated that although agreement is generally acceptable for individual CTG features, concordance markedly declines for overall trace classification, a limitation that has been linked to increased operative delivery rates and reduced diagnostic reliability [44]. In this context, cCTG offers advantages that extend beyond enhanced physiological sensitivity. Objective, algorithm-derived parameters such as STV, LTV, and acceleration frequency reduce subjectivity and improve reproducibility.

The present study supports this concept by showing that baseline FHR remained stable across physiological and pathological pregnancies, whereas variability and reactivity indices displayed clear, pathology-specific deterioration. These findings further emphasize the limited sensitivity of baseline FHR for detecting early or moderate fetal compromise and highlight the added value of dynamic cCTG indices in high-risk pregnancies.

The clinical relevance of reduced variability and attenuated acceleratory activity is supported by antepartum cCTG data from other settings. La Verde et al. reported that even in uncomplicated term pregnancies, prolonged maternal perception of Braxton–Hicks contractions was associated with reduced LTV and fewer accelerations, interpreted as reflecting cumulative uteroplacental flow reduction and early autonomic adaptation to transient hypoxemic stress [45]. Although derived from a low-risk population, these findings are directionally consistent with the alterations observed in the present high-risk cohorts, suggesting that diminished variability and reactivity represent a shared fetal autonomic response to diverse stressors.

Accordingly, the hierarchical pattern of autonomic impairment identified in this study—most pronounced in HDP and intermediate in GDM and ICP—appears physiologically coherent. Importantly, these alterations were detectable in the absence of baseline FHR abnormalities or frequent pathological decelerations, indicating that early fetal compromise is preferentially reflected in dynamic autonomic indices rather than static rate parameters. This observation aligns with the recognized limitations of visual CTG interpretation and supports the integration of multiparametric cCTG into antepartum surveillance strategies for high-risk pregnancies.

Several limitations should be acknowledged. The retrospective, single-center design may limit the generalizability of the findings. Moreover, this study was not designed to assess neonatal outcomes such as Apgar scores, umbilical arterial pH, lactate concentrations, or other biochemical markers of neonatal compromise. The primary aim was to provide a focused comparative analysis of antepartum cCTG parameters across distinct categories of high-risk pregnancies and physiological gestations, with an emphasis on fetal autonomic function and reactivity. As a consequence, neonatal clinical parameters and biochemical markers were not analyzed, and direct correlations between antepartum cCTG patterns and specific neonatal morbidity profiles fall outside the scope of the present investigation. This deliberate methodological choice allowed for a clearer characterization of cCTG-derived autonomic signatures associated with different high-risk pregnancy conditions.

## 5. Conclusions

This study demonstrates that antepartum quantitative cCTG analysis provides clinically meaningful, pathology-specific insights into fetal autonomic regulation in pregnancies complicated by HDP, GDM, and ICP. Compared with physiological pregnancies, high-risk cohorts exhibited consistent alterations in cCTG-derived indices of variability and reactivity, with marked reductions in STV, increased suppression of LTV below 5 bpm, and decreased acceleration frequency, particularly in HDP. These findings underscore the added value of quantitative cCTG metrics over baseline FHR for the early identification of autonomic dysregulation that may precede overt fetal compromise.

Interpretation of these findings should be framed within the broader clinical context. In routine obstetric practice, delivery decisions are based on an integrated assessment of maternal condition, fetal status, gestational age, and overall clinical evolution, rather than on cCTG parameters in isolation. Within this framework, the present results support the use of quantitative cCTG as an objective tool for identifying pathology-specific patterns of altered fetal adaptation and potential chronic hypoxemic stress.

From a clinical perspective, quantitative cCTG should be incorporated into multimodal surveillance strategies alongside Doppler velocimetry, fetal ECG-derived parameters, biochemical markers, and comprehensive maternal evaluation. Its relevance extends beyond the pathological conditions examined in this study. In clinical scenarios such as preterm or term premature rupture of membranes (PPROM/PROM) [46], where risks of infection, cord compression, and oligohydramnios necessitate intensified monitoring, quantitative cCTG may provide valuable adjunctive information by identifying subtle patterns of hypoxemic stress when conventional CTG findings are inconclusive.

Overall, these findings support the integration of quantitative cCTG into personalized monitoring protocols for high-risk pregnancies and provide a foundation for the development of multimodal, evidence-based surveillance pathways. The incorporation of advanced algorithm-based and machine-learning approaches may further refine risk stratification, enable real-time interpretation of fetal autonomic patterns, and enhance the precision of antenatal surveillance, ultimately improving early detection of fetal compromise and perinatal outcomes [47].

## Figures and Tables

**Table 1:** Baseline fetal heart rate (FHR) across study groups.

Parameter	Control (n = 87)	ICP (n = 86)	GDM (n = 146)	HDP (n = 169)
Minimum (bpm)	110	111	109	116
Maximum (bpm)	156	166	166	158
Median (bpm)	134	138	136	138
Mean (bpm)	135.4 a	136.5 a	136.4 a	137.7 a
SD (bpm)	9.5	8.8	9.9	8.3
COV (%)	7.05	6.46	7.26	6.04

Note: FHR—fetal heart rate; SD—standard deviation; COV—coefficient of variation. All groups exhibited baseline FHR values within the physiological range (110–160 bpm). Values connected by the same letter are not statistically different from each other (*p* > 0.05).

**Table 2:** Fetal movement count recorded by cCTG across study groups.

Parameter	Control (n = 87)	ICP (n = 86)	GDM (n = 146)	HDP (n = 169)
Minimum	0	0	0	0
Maximum	190	210	215	257
Median	27	45	59	65
Mean	37.0 a	55.9 b	66.4 b,c	74.5 c
SD	37.4	40.2	44.9	47.2
COV (%)	101.05	71.88	67.59	63.26

Note: Data is expressed as the number of fetal movements detected during cCTG recordings. Different letters indicate significant differences (*p* < 0.05) among means across different groups. Values connected by the same letter are not statistically different from each other.

**Table 3:** Percentage of time with short-term variability (STV) < 1 bpm across study groups.

Parameter	Control (n = 87)	ICP (n = 86)	GDM (n = 146)	HDP (n = 169)
Minimum (%)	31.00	34.00	34.00	37.00
Maximum (%)	80.00	81.00	97.00	97.00
Median (%)	53.00	57.00	58.00	64.00
Mean (%)	53.72 a	57.35 a,b	58.51 b,c	62.84 c
SD (%)	11.54	12.00	12.84	11.99
COV (%)	21.47	20.93	21.95	19.08

Note: STV—short-term variability; SD—standard deviation; COV—coefficient of variation. Higher STV values indicate longer periods of reduced fetal heart rate variability. Different letters indicate significant differences (*p* < 0.05) among means across different groups. Values connected by the same letter are not statistically different from each other.

**Table 4:** Average short-term variability (STV) across study groups.

Parameter	Control (n = 87)	ICP (n = 86)	GDM (n = 146)	HDP (n = 169)
Minimum (bpm)	0.60	0.60	0.20	0.30
Maximum (bpm)	2.60	2.20	2.10	2.00
Median (bpm)	1.30	1.20	1.20	1.00
Mean (bpm)	1.35 a	1.22 a,b	1.20 b,c	1.08 c
SD (bpm)	0.41	0.38	0.39	0.35
COV (%)	30.16	31.07	32.29	32.27
Range (bpm)	2.00	1.60	1.90	1.70

Note: STV—short-term variability; SD—standard deviation; COV—coefficient of variation. Lower average STV values indicate reduced fetal autonomic reactivity and diminished beat-to-beat variability. Different letters indicate significant differences (*p* < 0.05) among means across different groups. Values connected by the same letter are not statistically different from each other.

**Table 5:** Number of accelerations recorded by cCTG across study groups.

Parameter	Control (n = 87)	ICP (n = 86)	GDM (n = 146)	HDP (n = 169)
Minimum	0.00	0.00	0.00	0.00
Maximum	21.00	36.00	29.00	25.00
Median	6.00	4.00	5.00	3.00
Mean	6.78 a	5.56 a,b	6.12 a	4.21 b
SD	4.97	5.17	5.32	4.78
COV (%)	73.26	93.08	87.03	113.64
Times accelerations occurred	81	81	136	143
% of total patients	93%	94%	93%	85%

Note: Data represents the number of accelerations detected during cCTG recordings. Values indicate lower fetal reactivity in HDP pregnancies compared to control and other pathological groups. Different letters indicate significant differences (*p* < 0.05) among means across different groups. Values connected by the same letter are not statistically different from each other.

**Table 6:** Percentage of time with LTV < 5 bpm across study groups.

Parameter	Control (n = 87)	ICP (n = 86)	GDM (n = 146)	HDP (n = 169)
Minimum (%)	0	0	0	0
Maximum (%)	21	28	80	79
Median (%)	0	0	1	2
Mean (%)	2.8 a	3.4 a,b	5.4 a,b	6.8 b
SD (%)	5.0	6.0	12.0	11.0
COV (%)	179.50%	174.98%	221.72%	161.24%

Note: Higher values represent longer periods of reduced long-term fetal heart-rate variability. Different letters indicate significant differences (*p* < 0.05) among means across different groups. Values connected by the same letter are not statistically different from each other.

**Table 7:** Total Decelerations recorded by cCTG across study groups.

Parameter	Control (n = 87)	ICP (n = 86)	GDM (n = 146)	HDP (n = 169)
Minimum	0	0	0	0
Maximum	5	17	25	12
Median	0	0	0	0
Mean	0.31	1.09	0.92	1.19
SD	0.8	2.4	2.6	2.1
COV (%)	270.53%	222.62%	278.01%	177.62%
Times decelerations occurred	17	38	52	84
% of total patients	20%	44%	36%	50%

Note: There were no statistically significant differences among groups (*p* > 0.05).

**Table 8:** Repetitive Decelerations recorded by cCTG across study groups.

Parameter	Control (n = 87)	ICP (n = 86)	GDM (n = 146)	HDP (n = 169)
Times repetitive decelerations occurred	1	3	7	6
% of total patients	1%	3%	5%	4%

Note: There were no statistically significant differences among groups (*p* > 0.05).

**Table 9:** Late Decelerations recorded by cCTG across study groups.

Parameter	Control (n = 87)	ICP (n = 86)	GDM (n = 146)	HDP (n = 169)
Times late decelerations occurred	5	5	7	12
% of total patients	6%	6%	5%	7%

Note: There were no statistically significant differences among groups (*p* > 0.05).

**Table 10:** Prolonged Decelerations recorded by cCTG across study groups.

Parameter	Control (n = 87)	ICP (n = 86)	GDM (n = 146)	HDP (n = 169)
Times late decelerations occurred	1	3	3	6
% of total patients	1%	3%	2%	4%

Note: There were no statistically significant differences among groups (*p* > 0.05).

**Table 11:** Decelerations > 5 min recorded by cCTG across study groups.

Parameter	Control (n = 87)	ICP (n = 86)	GDM (n = 146)	HDP (n = 169)
Times decelerations >5 min occurred	1	1	3	4
% of total patients	1%	1%	2%	2%

Note: There were no statistically significant differences among groups (*p* > 0.05).

## Data Availability

The data underlying this study are not publicly available due to patient confidentiality and institutional data protection regulations. Aggregated or anonymized data may be made available from the corresponding author upon reasonable request and with permission of the Filantropia Clinical Hospital.

## Abbreviations

The following abbreviations are used in this manuscript:

Abbreviation	Meaning
cCTG	Computerized cardiotocography
CTG	Cardiotocography
FHR	Fetal heart rate
STV	Short-term variability
LTV	Long-term variability
HDP	Hypertensive disorders of pregnancy
GDM	Gestational diabetes mellitus
ICP	Intrahepatic cholestasis of pregnancy
PPROM	Preterm premature rupture of membranes
PROM	Premature rupture of membranes
ECG	Electrocardiography
ANOVA	Analysis of variance
SD	Standard deviation
COV	Coefficient of variation
bpm	Beats per minute
